# Ventriculomegaly without elevated intracranial pressure? Normal pressure hydrocephalus as a disorder of the cerebral windkessel

**DOI:** 10.3389/fneur.2025.1591275

**Published:** 2025-05-01

**Authors:** Racheed Mani, Jade Basem, Liu Yang, Nahid Shirdel Abdolmaleki, Anand Ravishankar, Susan Fiore, Petar Djuric, Michael Egnor

**Affiliations:** ^1^Department of Neurology, Stony Brook University Hospital, Stony Brook, NY, United States; ^2^Department of Neurological Surgery, Stony Brook University Hospital, Stony Brook, NY, United States; ^3^Department of Electrical and Computer Engineering, Stony Brook University, Stony Brook, NY, United States; ^4^Department of Anesthesiology, Perioperative and Pain Medicine, Stanford Medicine, Stanford, CA, United States

**Keywords:** normal pressure hydrocephalus, hydrocephalus, intracranial pressure, intracranial dynamics, pulsatility, mathematical modeling

## Abstract

**Objective:**

Normal pressure hydrocephalus (NPH) is characterized by ventriculomegaly without elevations in intracranial pressure (ICP). One way of viewing hydrocephalus is as a disorder of the cerebral windkessel. The cerebral windkessel is the system that dampens the arterial blood pressure (ABP) pulse in the cranium, transmitting this pulse from arteries to veins via the cerebrospinal fluid (CSF) path, bypassing the microvasculature to render capillary flow smooth. When the windkessel is physiologically tuned, windkessel effectiveness (*W*) is given by: *W*=*IE*/*R*, where *I* represents CSF path inertance (pulse magnitude), *E* is CSF path elastance, and *R* is resistance in the CSF path. In NPH, we posit that there is a combination of arteriosclerosis (blunting the CSF pulse in the SAS- lowering *I*), and age-related softening of brain tissue (decreasing the elastance of subarachnoid CSF pathways- lowering *E*).

**Methods:**

To model the windkessel, we utilize a tank circuit with parallel inductance and capacitance to simulate the pulsatile flow of blood and CSF as alternating current (AC), and smooth flow as direct current (DC). We model NPH as a disorder of windkessel impairment by decreasing windkessel inertance (reflecting diminished CSF pulsatility in the SAS from arteriosclerosis) and decreasing intracranial elastance (reflecting age-related brain atrophy). We simulate ventriculomegaly and shunting by lowering the resistance of this circuit.

**Results:**

In simulating NPH using this circuit, we found significant elevations in the amplitude and power of AC in the CSF and capillary paths when inertance and elastance were decreased. Conversely, this pulse power decreased with decreased resistance in the CSF path from ventriculomegaly and shunting.

**Conclusion:**

Simulations of NPH demonstrated increased amplitude and power of AC in the CSF and capillary paths due to windkessel impairment. We posit that this pulsatility is redistributed from the SAS to the ventricular CSF path, exerting pulsatile stress on the periventricular leg and bladder fibers, which may explain NPH symptomatology. Ventriculomegaly may represent an active adaptation to improve windkessel effectiveness by decreasing CSF path resistance to mitigate decreased CSF path inertance and parenchymal elastance. Shunting provides a low resistance, accessory windkessel to obviate adaptive ventriculomegaly. This has significant implications in understanding this paradoxical condition.

## Introduction

Normal pressure hydrocephalus (NPH), first defined in 1965 by Drs. Salamon Hakim and Raymond Adams, is characterized by the triad of gait disturbance, cognitive impairment, and urinary incontinence. It is also distinguished by the apparent paradox of ventriculomegaly *without* significant elevations in intracranial pressure (ICP) (1). NPH is diagnosed based on combinations of the symptoms above (most commonly presenting with gait disturbance) (2–4), together with imaging demonstrating ventricular dilatation (an Evans’ index greater than 0.3) out of proportion to age-related parenchymal atrophy, without obstructive lesions or other organic etiologies of hydrocephalus (4–6). Often referred to as “disproportionately enlarged subarachnoid space hydrocephalus” (DESH), this condition is often characterized by other radiographic findings such as dilated Sylvian fissures with focal dilation of sulci (‘skipped’ sulci), gyral crowding near the convexity (7), acute callosal angle, temporal horn dilation disproportionate to hippocampal atrophy, and bowing of the corpus callosum (2–4). The diagnostic probability of the condition further increases following ‘tap testing’, with lumbar punctures (LPs) yielding with normal opening pressures (5–18 mm Hg) and commonly an improvement in symptoms following large volume taps (2, 4). The definitive treatment for NPH patients remains cerebrospinal fluid (CSF) shunting, with clinical improvement noted in up to 84% of patients diagnosed with the condition (2, 4, 8).

Various theories have been proposed to account for the pathophysiology of NPH and its apparent paradox of ventriculomegaly with little or no elevation in ICP (4, 9). These theories analyze NPH as a disorder of the cerebral vasculature, with arteriosclerosis, reduced intracranial elastance, and impaired glymphatic clearance among the disease processes implicated (4, 9). Part of the challenge in understanding the disease process is that the Monro-Kellie pressure-volume model, from which our fundamental understanding of hydrocephalus is derived (10–12), does not account for many of the clinical and experimental characteristics associated with different forms of hydrocephalus, particularly NPH. The Monro-Kellie framework is centered on mass conservation, stating that the cranium consists of brain, blood, and CSF, and any change in one component is accompanied by a commensurate change in the others (10–12). In this framework, hydrocephalus is understood to be a disorder of ‘bulk flow’ of CSF, with an imbalance between the formation and reabsorption of CSF, a pathological process *not* at play in NPH.

### Hydrocephalus as a disorder of intracranial pulsatility

Considering the inherent limitations of the ‘bulk flow’ model, another approach to hydrocephalus has been to view the condition as a disorder of intracranial pulsatility (13–16). There is substantive literature demonstrating that, rather than being a disorder of the ‘back up’ of CSF, hydrocephalus is a clinical manifestation of the redistribution of pulsatility from the subarachnoid space (SAS) to the ventricles (9, 17–20).

Investigations have demonstrated that hyperdynamic choroid plexus pulsations are necessary and sufficient for ventricular dilation in communicating hydrocephalus even without any obstruction to CSF flow (17–21). Moreover, unilateral choroid plexectomy and interruption of flow from the ipsilateral anterior choroidal artery prevented the dilation of the ipsilateral ventricle (but not the contralateral ventricle). Conversely, intraventricular infusions of saline (to simulate the ‘bulk flow’ model) did not induce ventriculomegaly (22). Others have noted that communicating hydrocephalus could be induced by increasing the amplitude of the intraventricular pulse pressure even while keeping the mean CSF pressure and absorption constant (19).

As part of normal physiology, these pulsations are transmitted from the arteries to the cranium during each cardiac cycle. This arterial blood pressure (ABP) pulse is dampened in the cranium by transmitting this pulse from arteries to veins via the CSF pathway, bypassing the microvasculature to render capillary flow smooth (14–16, 23). This pulsatile CSF flow has been demonstrated on flow MRI studies, showing that pulsatile arterial, CSF, and venous flow are normally synchronous throughout the cardiac cycle (20). The CSF path, therefore, may serve as a hydraulic link of arterial expansion and relaxation to venous compression and re-expansion. This has been proposed as the ‘cerebral windkessel’ mechanism (15, 16).

### The cerebral windkessel and the separation of DC and AC power in the cranium

We have proposed that the cranium is perfused by a pulse pump, with energy going in and out of the cranium through arterial and venous blood flow comprised of smooth power and pulsatile power (15, 16). This stems from the observation that the cranium acts as a band-stop filter centered at the heart rate for the intracranial pressure (ICP) pulse in relation to the arterial blood pressure (ABP) pulse, with the ICP pulse preceding the ABP pulse in a normal physiological state (15, 16, 23).

**Figure 1:**
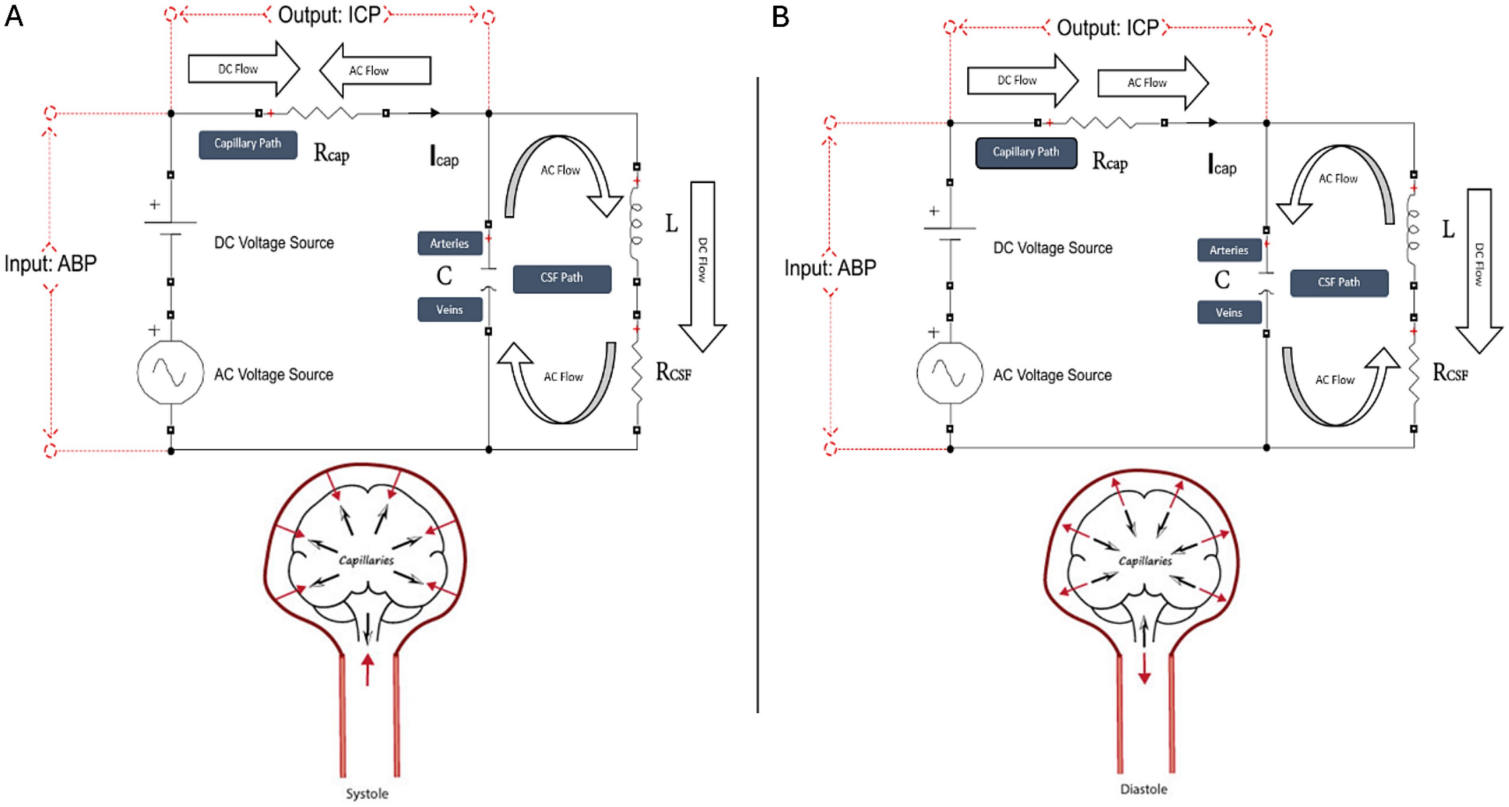
Schematic of our tank circuit model simulating systole **(A)** and diastole **(B)**. The tank circuit has a DC and AC source, a series resistor R_cap_ (corresponding to the capillary path), and a parallel RLC circuit (corresponding to the CSF, interstitial fluid, brain parenchyma, and intracranial arteries and veins, which, for brevity, we call the CSF path). Voltage represents pressure, current represents flow, and charge represents volume. Voltage across the source is a proxy for ABP, and voltage across the capillary resistor is a proxy for ICP. Capacitance (C) represents the compliance of the extracapillary (parenchymal and CSF) path and corresponds primarily to the compliance of the intracranial veins. Inductance (L) represents intracranial inertance, which corresponds to the inertance of the extracapillary cranial contents. R_CSF_ represents structural damping of the vascular walls and brain parenchyma and resistance to compression/expansion of the cerebral arteries and veins. R_CSF_ is effectively a venous pump. R_cap_ corresponds to intracapillary resistance to longitudinal blood flow, and R_CSF_ is the net resistance in the extracapillary space to radial expansion and relaxation of capillary walls. Note that R_CSF_ does not represent the resistance to CSF absorption. The model clarifies the dynamics of the cerebral windkessel. Suppression of the AC current from the capillary path is accomplished by cyclic loading and unloading of the ‘arterial’ and ‘venous’ capacitor plates, which continuously opposes and blocks the cyclic current from the AC arterial source. In systole **(A)**, caudal motion of the brain parenchyma opposes cranial motion of the arterial pulse entering at the skull base. The systolic arterial pulse is depicted in the figure by red solid arrows, and the centrifugal and caudal brain motion is depicted by black arrows. In systole, brain expansion and caudal brain displacement deflects a portion of the systolic arterial pulse away from the capillary circulation and diverts the pulsatile energy through the CSF spaces to the veins. Systolic centrifugal motion of the parenchyma opposes centripetal inflow of the arterial pulse in the perforating arteries, and radial inward compression by the expanding brain parenchyma opposes radial outward expansion of the capillary walls. In diastole **(B)**, cranial rebound of the brain (black arrows) opposes diastolic caudal regress of the arterial pulse (red arrow) at the skull base. Diastolic centripetal motion of the cortex (black arrows) opposes diastolic centrifugal regress of the arterial pulse (red arrows) in the perforating arteries, and centrifugal relaxation of the parenchyma opposes centripetal relaxation of the capillary walls.

In our model, smooth blood flow is the equivalent of the direct current (DC) offset and corresponds physiologically to smooth mean cerebral blood flow (CBF), and pulsatile flow represents the alternating current (AC) flow and corresponds physiologically to pulsations of blood, CSF, and brain parenchyma. A schematic of our model simulating intracranial dynamics in systole and diastole is demonstrated in Figure 1 (15, 16).

In normal physiology, nearly all blood flow in the cerebral capillaries is DC power, as the presence of AC power in microvasculature risks capillary disruption and parenchymal edema (15, 16). Conversely, nearly all CSF flow is AC power, with flow MRI imaging indicating that the maximal AC kinetic energy of the CSF pulsations at the level of the cervicomedullary junction is 100,000 times greater than the DC kinetic energy of CSF bulk flow in the cerebral aqueduct (15, 16). We proposed that the CSF path serves as a hydraulic linkage to transmit AC power from the arteries to the veins, thereby bypassing the microvasculature to protect the capillaries from this pulsatility. CSF links arterial expansion and relaxation to venous compression and re-expansion. MRI studies in humans reveal expansion and relaxation of the cranial contents during the cardiac cycle that continuously opposes arterial expansion and relaxation, and we have suggested that this is the way the windkessel is implemented in the cranium (14, 15, 23) This separation of AC and DC power is the windkessel effect, with and the windkessel is a cornerstone of intracranial thermodynamics serving to optimize DC power and minimize AC power in capillary blood flow (16).

When the windkessel is physiologically tuned, windkessel effectiveness (*W*) is given by:

W=IE/R

where *I* is the CSF path inertance (i.e., pulse magnitude), *E* is CSF path elastance, and *R* is resistance in the CSF path and the damping in the brain parenchyma (15, 16).

Perturbations to the mechanism above which may lead to redistribution of that pulse pressure may be implicated in various forms of hydrocephalus, including NPH. Egnor and colleagues have proposed that various forms of hydrocephalus are due to impairments of windkessel effectiveness (14–16, 23).

Windkessel impairment can be due to increased impedance due to increased resistance in the CSF spaces to CSF pulsations (as seen in obstructive hydrocephalus) or to increased impedance in the CSF spaces due to decreased inertance (arteriosclerosis) and decreased elastance (brain atrophy) (16).

We have proposed that ventriculomegaly is an active adaptation to hydrocephalus and serves to decrease $R_{CSF}$ to improve windkessel effectiveness (15, 16). Looking at CSF path as a cylindrical tube, we have proposed previously that $R_{CSF}$ can be given by Poiseuille’s law based on prior work using this concept to represent resistance to flow (15, 16, 24):

$$R_{CSF}=\frac{8\eta l}{\pi r^{4}}$$

where r represents the radius of the CSF space (reflecting the ventricles), l is the length of the (cylindrical) CSF space, and η is CSF viscosity.

In NPH, there is a combination of arteriosclerosis (which blunts the CSF pulse magnitude in the SAS- lowering *I* in the windkessel equation), and age-related softening of brain tissue as noted by findings from magnetic resonance elastography (MRE) (which decreases the elastance of subarachnoid CSF pathways- lowering *E* in the windkessel equation) (15, 16, 23). These, in turn, reduce windkessel effectiveness.

Ventricular dilatation, through decreasing R_CSF_, would thereby increase the volume of available CSF for coupling of arterial to venous pulsations, an active, adaptive response to mitigate windkessel impairment, not a passive process (14–16). This explanation may account for the apparent paradox of ventriculomegaly even without elevated ICP. Therefore, CSF diversion via shunting may provide a long-term means of lowering resistance in this CSF path by providing an additional conduit for this pulsatility. Shunting therefore provides an accessory windkessel, thus lowering the need for adaptive ventriculomegaly (14–16).

To demonstrate this theory, we seek to use an electrical tank circuit model to simulate the cerebral windkessel and introduce perturbations by reducing elastance (*E*) and inertance (*I*) to simulate NPH. We also simulate how ventriculomegaly serves as an initial adaptation to reduce R_CSF_ and how shunting is a curative modality to reduce R_CSF_ in this condition.

## Methods

### The basis of our cerebral windkessel model

To model the cerebral windkessel, we utilize an electrical tank circuit, the simplest representation of a band-stop filter, with parallel capacitance and inductance based on our prior work (15, 16). In our model, we compared the circuit dynamics to physiological ICP and ABP data from 12 dogs (18–26 kg) through autoregressive with exogenous inputs (ARX) modeling (16, 23). In this model, ABP data represented the input to the system and the ICP data the output (15, 16).

Our study at that time was approved by our Institutional Animal Care and Use Committee (IUCAC), and the animals were treated humanely in accordance with the Guide for the Care and Use of Laboratory Animals published by the National Institutes of Health (NIH). The 12 dogs received sedation with thiopental, were anesthetized with 1.5% isoflurane, and intubated. To obtain ABP readings, a 1 mm Microtip pressure transducer (SPR-524 Millar Instruments) was placed into the right common carotid artery in each canine. To obtain ICP data for each dog, a right frontal burr hole was drilled, and the dura mater was opened, with a Microtip catheter inserted to a depth of 1–2 cm into the brain parenchyma. Data was collected for approximately 15 min in the resting state.

In this circuit, smooth mean cerebral blood flow (CBF) is the equivalent of the direct current (DC) offset, and alternating current (AC) flow corresponds to the pulsatility of blood, CSF, and parenchyma. In our published model, we found a very close correspondence (to within 2.5% error) between the tank circuit and the pulse suppression in from the canine data, validating our model as an appropriate engineering analog with which to simulate the dynamics of the cranium (15, 16). We provide a detailed outline of ARX modeling and the derivation of the windkessel effectiveness equation in the Supplementary material of our published work (16). Our circuit model is outlined in Figure 1 (15, 16).

In this model, voltage corresponds to pressure, current corresponds to flow and motion, and charge to the displacement of fluid and tissue. Inductance corresponds to the size of the pulse, capacitance represents parenchymal compliance, and resistance represents intracranial damping.

We started our simulation with the following estimated physiological parameters to represent normal intracranial dynamics:

Resistance of the capillary path (*R*_cap_) = 18.6085 Ohm (*Ω*)

Resistance of the CSF path (*R*_CSF_) = 225.1457 Ω

Inertance (*I*) = 126.9883Pas^2^m^−3^

Elastance (*E*) = 3333F^−1^

### Simulation of NPH using our tank circuit model

We simulate the pulsatile flow of blood and CSF as alternating current (AC), and smooth blood flow as direct current (DC). We monitor the instantaneous power of AC in both the capillary and CSF paths.

After initially simulating normal intracranial dynamics, at 10 s (s), we introduce the following perturbations to simulate NPH: increasing compliance two-fold (to simulate decreased parenchymal elastance) and decreasing the intracranial inertance two-fold (to simulate arteriosclerosis decreasing the pulse in the CSF path). Foltz et al. (25) found that the power of intraventricular pulsations was increased up to 4-fold in chronic hydrocephalus. Therefore, to replicate this 4-fold alteration in the intracranial dynamics, we have opted to alter the windkessel effectiveness equation by increasing compliance and decreasing inertance each by a factor of two.

At 20s, we decrease the resistance within the CSF path two-fold to simulate the ventriculomegaly seen in NPH (15, 26).

At 30s, we simulate the effect of CSF shunting (by decreasing the resistance in the CSF path further by a factor of two- corresponding to a four-fold decrease from baseline) (15, 26).

## Results

During normal physiology with preserved windkessel function, the power of AC flow in the capillary path is negligible. However, in a pathological state such as NPH, after decreasing inertance (*I*) and elastance (*E*) to lower windkessel effectiveness by a total factor of 4 to simulate NPH, we noticed significant increases in the amplitude of the instantaneous power of AC flow in the CSF path as well as in the capillary path (albeit to a lesser extent than the CSF path).

In lowering the resistance within the circuit by a factor of two (to simulate lowering resistance in the CSF path to reflect the process of ventricular dilatation), we noted decreases in this instantaneous power in the CSF and capillary paths, albeit not back to baseline levels during normal physiology. Ventriculomegaly, through increasing the radius of the CSF pathways, partially lowers resistance and partially reduces power.

**Figure 2:**
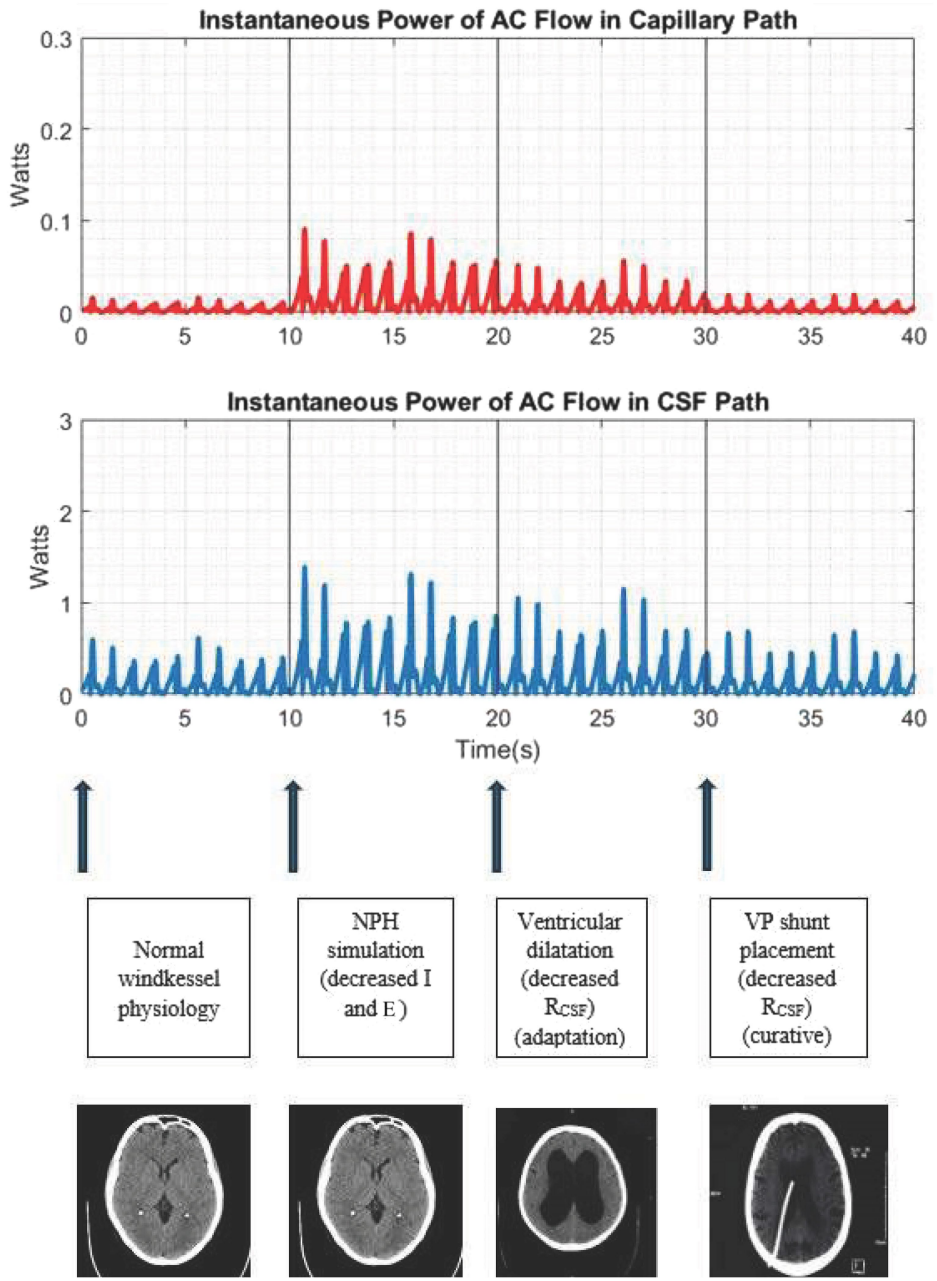
Simulation of the cerebral windkessel and perturbations to simulate NPH. At 0–10s, we demonstrate normal physiology, with negligible AC flow in the capillary path. At 10s, we decrease the intracranial inertance (*I*) and elastance (*E*) to simulate NPH, noting an increase in the amplitude of AC power in both the CSF and capillary paths. At 20s, we decrease resistance in the CSF path (a proxy for ventricular dilatation), which lowers the amplitude of AC power, albeit not to baseline. At 30s, we further lower resistance of the CSF path to simulate CSF shunting, a definitive treatment which provides another accessory windkessel and returns the intracranial pulse distribution to the physiological baseline.

We then demonstrated CSF shunting as a means of providing a therapeutic conduit for this AC power via a lower resistance path (thereby lowering *R* in the windkessel effectiveness equation). When lowering the resistance in the CSF path further by a factor of two (corresponding to a total four-fold decrease in *R*) to simulate CSF shunting, we noted a reduction in the AC power in both the CSF and capillary paths back to baseline. These results are shown in Figure 2.

## Discussion

The cerebral windkessel mechanism is the suppression of the arterial blood pressure (ABP) pulse in the cranium via the rhythmic motion of the brain during systole and diastole that opposes the ABP pulse (Figure 1) (15, 16). This windkessel relies upon the coupling of arterial expansion and venous compression using the CSF path as a hydraulic linkage. The energy of cerebral blood flow (CBF) entering the cranium consists of AC (pulsatile flow) and DC (bulk flow). The cerebral windkessel allows for the diversion of AC power from the arteries to the veins via the CSF path, bypassing the microvasculature to render capillary flow smooth, while allowing for capillary perfusion with DC power (15, 16). The cranium is a series of pathways through which energy flows, and the flow of this energy is power. We propose that the cerebral windkessel is the cornerstone of intracranial thermodynamics, and that this framework, as opposed to the ‘bulk flow’ model, is one through which we should analyze disorders of intracranial dynamics going forward.

In our model, hydrocephalus can be viewed as windkessel impairment due to increased impedance to AC power in the CSF path, thus diverting more of this AC power to the capillary path. We posit that various forms of hydrocephalus are reflections of perturbations to one or more variables in the equation for windkessel effectiveness (*W=IE/R*) (15, 16).

Our simulation of NPH demonstrated increased amplitude of power of the AC pulse in the capillary path. The increase in power of the AC in the CSF path represents the transfer of the pulse in the subarachnoid path to the ventricular CSF spaces due to the reduction of the pulse in the SAS. This blunting of the CSF pulse reflects decreased intracranial inertance (*I*), which manifests due to the arteriosclerosis which characterizes the cerebral vasculature in NPH (27, 28). NPH is also characterized by softening of brain tissue, corresponding to decreased parenchymal elastance (lower *E*).

The dynamics of windkessel impairment in NPH differ from the dynamics of high CSF path resistance (increased R) which would characterize obstructive hydrocephalus (OH). In high resistance windkessel impairment (i.e., OH), the circulation current (i.e., absorber brain motion) is ‘glued’ and windkessel impairment is more deleterious, with marked redistribution of AC power to capillaries. This alteration in thermodynamics would manifest with consequent edema and capillary disruption that may be life-threatening. We propose that this accounts for why OH has a much more severe clinical presentation than NPH.

Conversely, windkessel impairment due to decreased elastance, as seen in NPH, is less deleterious to capillaries. In NPH, the CSF path spaces are still open to serve as a conduit for intracranial pulsatility. The pulsatile stress is less on the capillary walls than in OH, but is diverted more to the ventricular CSF and to the periventricular parenchyma. The windkessel is able to protect capillary walls, albeit at the price of higher pulsatile stress on the leg and bladder pathways adjacent to the ventricles. The pulsatile brain tissue stress—which corresponds to the circulation current that accomplishes the windkessel—is proportional to capillary wall motion and inversely proportional to resistance in the CSF path (14, 15).

In NPH, a higher amplitude of pulsatility at the level of the ventricular CSF (corresponding thermodynamically as increased kinetic energy of AC in the CSF path) is required to facilitate functioning of the windkessel. This manifests as increased intraventricular CSF oscillations exerting greater pulsatile stress on the periventricular white matter tracts corresponding to the lower limb and bladder, which may account for the symptoms of gait apraxia and urinary incontinence, respectively.

Ventriculomegaly may represent an adaptive response to improve windkessel effectiveness by decreasing CSF path resistance, which, in the case of NPH, mitigates decreased parenchymal elastance (from atrophy) and decreased inertance (from arteriosclerosis). This was simulated using our model, with a reduction in Rcsf reducing the amplitude of the instantaneous power in the capillary and CSF paths, albeit not to baseline.

Following this model, CSF diversion via shunting can be viewed as a conduit to divert energy through a low resistance pathway and dampen the ventricular CSF pulse. This reduces the resistance in the windkessel effectiveness equation and thereby improves windkessel effectiveness. As our simulation demonstrates, this reduction in resistance (*R*) reduces the amplitude of instantaneous power in both the capillary and CSF paths to baseline levels. This would thereby reduce the pulsatile strain on leg and bladder tracts and is ultimately therapeutic in restoring normal intracranial thermodynamics. Shunts can therefore be seen as an accessory windkessel to drain energy, thereby reducing the need for adaptive ventriculomegaly.

### Limitations and future directions for investigation

As we highlight in our prior work (16), our model faces the inherent limitation of using an electrical engineering analog to simulate physiologic dynamics and parameters within the cranium. Physiologically, these parameters within the cranium (elastance, inertance, and resistance) are specific to the radial motion of capillary walls, which have hitherto never been measured. Nevertheless, there is continuity between the windkessel in the aorta described by Frank (29) and the cerebral windkessel, and we set the tank circuit simulation to correlate with resistance, inertance, and capacitance values as measured by others who have analyzed the aortic windkessel (29, 30). Moreover, the close correlation (within 2.5% error) of our model to ABP and ICP data from dogs using ARX modeling demonstrates the validity of our model and approach.

Additionally, our model does not account for perturbations in venous hemodynamics, which authors such as Bateman have noted in NPH patients (31). Bateman has observed reduction in venous compliance with elevations in venous pressure in the area of the superior sagittal sinus (SSS) in NPH patients. He specifically cites a reduction in *superficial* venous drainage in NPH (31). We propose that venous hypertension may itself serve as an adaptation to increase intracranial elastance (E) to mitigate the softening of parenchyma seen in NPH, thereby improving effectiveness of the windkessel. Venous hypertension may also be the consequence of the windkessel impairment compromising the CSF venous pump, causing venous stasis and venous hypertension. (14, 15)

Furthermore, another area implicated in NPH is that of impaired glymphatic clearance, which is not addressed in this simulation. Iliff et al. proposed that cerebral arterial pulsatility is what drives paravascular CSF-interstitial fluid exchange of the glymphatic system (32). Perturbations to this glymphatic system have been implicated in various neurodegenerative conditions due to the accumulation of products such as amyloid-beta (Aβ) (33–35). Reductions in aquaporin (AQP4) expression in the astrocytic foot processes have been observed in brain tissue in NPH and Alzheimer’s Disease (AD) patients (33–35). In NPH, radiographic studies have demonstrated impaired glymphatic flow in NPH patients, as noted by decreased clearance of intrathecal gadolinium from the SAS.

We propose that the glymphatic system and the windkessel are interconnected, in that the pulsatile energy at the arterial perivascular space may be greater than the pulsatile energy at the venous perivascular space, and that there may be a pulsatile power gradient which may drive glymphatic flow. We propose that this explanation allows us to connect the two mechanisms and propose the ‘windkessel-glymphatic theory’ as a broadly encompassing framework through which to understand intracranial dynamics. Further investigation using magnetic resonance imaging (MRI) studies to analyze arterial and venous blood flow and pulsatility may provide greater insight into how the windkessel mechanism is both interconnected with and may account for the dynamics that drive the glymphatic system.

### Future considerations for treatment

Viewing NPH (and other forms of hydrocephalus) as disorders of intracranial thermodynamics opens another avenue of further inquiry: investigating novel approaches to treating NPH.

Since CSF shunting remains the definitive treatment for hydrocephalus, shunt designs should take into account pulsatile dynamics. The notion that NPH is a disorder of windkessel impairment causing redistribution of the intracranial pulsatility from the SAS to the ventricle provides another avenue of inquiry: that of CSF diversion to return the intracranial pulsatility to the SAS. The development of CSF shunting procedures was pioneered by a ventriculocisternal shunt known as the Torkildsen shunt (35). First performed by Norwegian neurosurgeon Dr. Arne Torkildsen in 1937, this procedure diverts CSF from the lateral ventricle to the cisterna magna (36, 37). Initially indicated for hydrocephalus in the setting of aqueductal, third, or fourth ventricular obstruction, this approach fell out of favor due to the development of ventriculoperitoneal (VP) and ventriculoatrial (VA) shunts (37). However, this type of shunt, in theory, allows for a conduit to directly re-transfer the AC power from the ventricles back to the SAS where the intracranial pulsatility is transmitted in normal physiology. Moreover, as a catheter bridging two CSF spaces, it does not require a valve to control drainage pressure. This may mitigate the risk of over- or under drainage (36). It is worth investigating for a possible role for this type of shunt in NPH patients based on the framework of the cerebral windkessel and (re) distribution of the intracranial pulsatility.

Furthermore, our model allows for the investigation of non-surgical interventions for NPH. Firstly, therapeutic cerebrovenous hypertension is already used in management of low-pressure hydrocephalus (via neck wrapping and resulting jugular depression) to mitigate reduced brain turgor/intracranial elastance. This may also be beneficial for NPH, a condition also characterized by reduced intracranial elastance. These are all avenues that should be explored further.

## Conclusion

To conclude, simulations of NPH demonstrated increased amplitude of AC pulse power in the capillary and CSF paths due to windkessel impairment. We posit that, in NPH, this pulsatile stress is delivered to the periventricular leg and bladder fibers, which may explain the symptomatology in NPH. Ventriculomegaly may represent an adaptive response in both conditions to improve windkessel effectiveness by decreasing CSF path resistance, which, in the case of NPH, mitigates decreased parenchymal elastance and decreased intracranial inertance. CSF shunting therefore may serve as an accessory windkessel through providing a low-resistance conduit for the AC pulse power. This framework has significant implications in our understanding and approach to treating NPH.

## Data Availability

The original contributions presented in the study are included in the article/supplementary material, further inquiries can be directed to the corresponding author.

## Author’s note

Mani R. Yang L, Djuric P, Fiore S, Egnor M. Ventriculomegaly Without Elevated Intracranial Pressure? Normal Pressure Hydrocephalus as a Disorder of the Cerebral Windkessel. AANS Annual Scientific Meeting, April 2023, Los Angeles, CA, United States.

## Glossary

NPH: Normal Pressure Hydrocephalus
ICP: Intracranial Pressure
ABP: Arterial Blood Pressure
CSF: Cerebrospinal Fluid
SAS: Subarachnoid Space
AC: Alternating Current
DC: Direct Current
LP: Lumbar Puncture
DESH: Disproportionately Enlarged Subarachnoid Space Hydrocephalus
MRI: Magnetic Resonance Imaging
MRE: Magnetic Resonance Elastography
ARX: Autoregressive with Exogenous Inputs
NIH: National Institutes of Health
IUCAC: Institutional Animal Care and Use Committee
OH: Obstructive Hydrocephalus
SSS: Superior Sagittal Sinus
Aβ: Amyloid-beta
AD: Alzheimer’s Disease
VP: Ventriculoperitoneal
VA: Ventriculoatrial
